# Decoherence in spin wave propagation via precursor pulses during signal equilibration

**DOI:** 10.1038/s41598-025-88799-3

**Published:** 2025-02-06

**Authors:** Cameron Aidan McEleney, Robert E. Camley, Rair Macêdo

**Affiliations:** 1 James Watt School of Engineering, Electronics & Nanoscale Engineering Division, University of Glasgow, Glasgow, G12 8QQ UK; 2 Department of Physics and Energy Science, Center for Magnetism and Magnetic Materials, University of Colorado at Colorado Springs, Colorado Springs, CO 80918 USA

**Keywords:** Numerical simulation, Solution of equations, Dispersion relations, Decoherence, Spin chain models, Spin waves, Ferromagnetic resonances, Electronic and spintronic devices, Physics, Ferromagnetism, Magnetic properties and materials, Spintronics, Atomistic models, Magnetic properties and materials, Spintronics

## Abstract

The processes for generation, amplification, and protraction of oscillating signals—often for information transfer—are inherently associated with dispersive decoherence and nonlinear phenomena. One such example is the case of wave patterns of non-negligible amplitudes that can emerge due to dispersion of a signal propagating through matter; for a continuously applied drive these patterns precede the main signal. Here, we investigate how spin wave generation inherently results in dispersive decoherence in the form of precursors. By quantifying three different frequency regimes, we investigate how decoherence is affected, or predetermined by the shape of the spin wave dispersion relation and, what is perhaps most interesting, it does not require non-linearity. Understanding the relationship between spin wave dispersion and decoherence can enable engineering magnonic devices supporting well-resolved signals for magnonic computing and signal processing.

## Introduction

Efforts to advance the mechanisms through which we transfer information have been the subject of intensive research efforts in recent years^1–3^. Despite the broad range of proposed ideas being brought forward, one often finds several fields grappling with similar challenges—that of efficient signal control and manipulation^4^. This is due to the common behaviours and characteristics of the oscillatory, dynamic waves which typically act as the information carrier^5^. Such transfer relies upon coherent, periodic signals which both are long-range and stable. Although these behaviours are well understood for electronic-based implementations of information transfer, translating these features into other platforms is an active area of research. One such possible platform is spintronics^6^, i.e. using spin waves, as they offer low intrinsic energies^7^, rapid signal transmission^8,9^, and support high operation frequencies that are essential for spin-based computating applications^10^. Furthermore, spin waves are able to transmit information without incurring joule heating losses. Spin waves are also inherently nonlinear in certain instances^11,12^; offering further adaptability to system designs.

There are, however, specific challenges to the manipulation of spin waves that can affect signal information transfer^7^. Spin waves are highly dependent upon the used medium which can lead to low-amplitude, decoherent signals^13^ propagating for only a handful of microns^14^. A related problem is in finding methods to generate high frequency magnons^15–17^ which can propagate for large distances^8,18^. Compensating for these effects, and others, has led to the pursuit of new spintronic mediums and novel, more efficient ways to drive these mediums and generate spin waves^6^. In many instances, efforts to amplify and protract the original signal lead to the generation of other unusual phenomena.

One such phenomenon is the recent generation and observation of dispersive shock waves (DSW)^19^—and their inherent features alongside, or instead of, the desired signal—which were interpreted using nonlinear descriptions^11,20–22^. DSWs occur in dispersive media, forming due to the resolution of a discontinuity^23,24^ arising during a gradient catastrophe^25^. DSWs have been both mathematically studied^25,26^ and identified in a variety of physical systems including: water waves^27,28^, intense laser light^29^, Bose–Einstein condensates^30^, ultra-cold atoms^31^, and between viscously contrasting fluids^32^.

Here we investigate phenomena that arise in generating spinwave signals with attention to data transfer or signal manipulation. In particular, we concentrate on the existence and properties of precursors and the time that it takes for the main signal onset to arrive. We find that at low frequencies the precursors can extend over several nanoseconds and, in addition, even when the main signal arrives it can take another few nanoseconds to achieve a steady state status. We show that at higher frequencies or in antiferromagnets many of these issues disappear or are reduced. Finally, we note that our results are similar to those found in DSW, even though our calculations are done in the linear limit of small amplitude signals. We are not suggesting that earlier studies are incorrect, but are simply pointing out that simpler models can produce signals that are reminiscent of the DSW results.

We show how the dispersion relation of a ferromagnetic system, resembling yttrium iron garnet (YIG), can be used to control or (and) predict which type of decoherence is observed. In doing so, we uncover analogues to the evolutionary properties of light’s precursor fields that are an intrinsic characteristic of linear propagation of step-modulated light pulses in dispersive media^33^. In particular, we find emergent pulse-like precursors that are counterparts to both Sommerfeld and Brillouin ‘*forerunners*’^34^—successive transients preceding the establishment of the steady-state pulse^35^—that were first conceptualised to remove inconsistencies between classical theory of waves and special relativity^36^.

To investigate spin wave propagation, we constructed a simple one-dimensional spin chain model comprising *N* sites. Each site *n* has a normalised magnetic moment $\pmb {m}_{\text {n}}$. The damped equation of motion for the magnetisation at a given site is governed by the standard Landau–Lifshitz–Gilbert (LLG) equation^37–39^:

1 $$\begin{aligned} \frac{d \pmb {m}_{n}}{dt}= -\mu _0 |\gamma |(\pmb {m}_n \times \pmb {H}_{\text {eff}} ) + \mu _0 \alpha |\gamma | [\pmb {m}_n \times (\pmb {m}_n \times \pmb {H}_{\text {eff}} )], \end{aligned}$$

where $\gamma$ is the gyromagnetic ratio, $\mu _0$ is the permeability of free space, and $\alpha$ is the Gilbert damping coefficient. The effective field $\pmb {H}_{\text {eff}}$ at site *n* is described by

2 $$\begin{aligned} \pmb {H}_{\text {eff}} = H_0 \pmb {\hat{z}} + \frac{J}{\mu _0} (\pmb {m}_{n-1} + \pmb {m}_{n+1} ) + \pmb {h}_d, \end{aligned}$$

where the first, second, and third terms are the externally applied magnetic field, exchange field, and driving field respectively.

Here we neglect dipolar effects due to dominance of exchange interactions at high frequencies^40^. Evidence of the negligible impact of long-range dipolar interactions in our model can be seen in Supplementary Note S1. Therefore, all interactions are mediated by the short-range exchange interactions between adjacent sites. For what follows, we also assume that the magnetisation dynamics are always in the linear regime, and thus, $m_n^x (t)$ and $m_n^y (t)$ are small and proportional to $e^{- \textrm{i} \omega t}$ while $m_n^z \approx 1$.

**Fig. 1:**
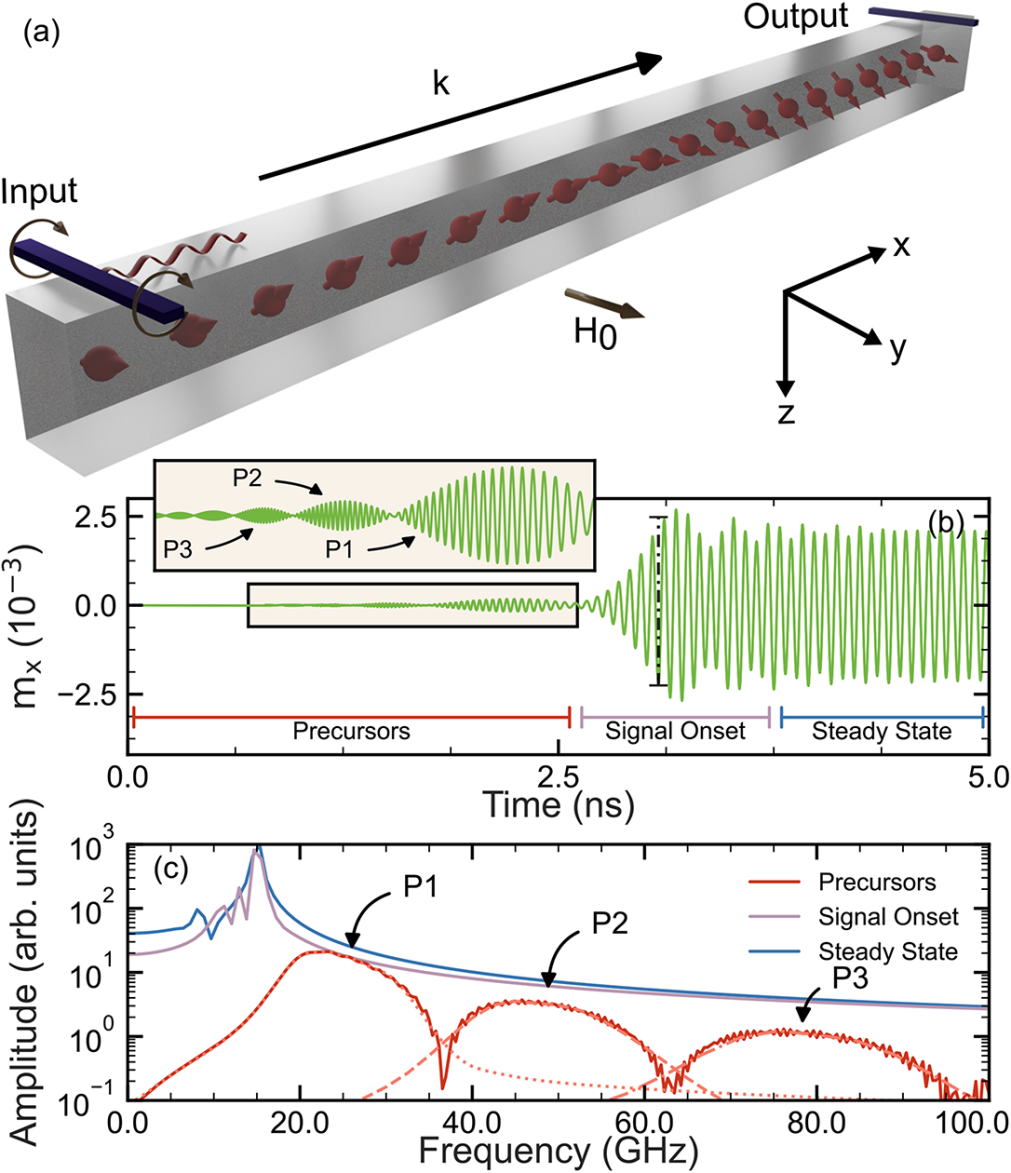
(**a**) Schematic of the mechanism for spin-wave generation from one side of a 1D spin chain and measured at the opposite end. (**b**) Time evolution of a signal at $f_d=$ 15 GHz, where the dashed line indicates the arrival time for a signal with frequency $f_d$, and (**c**) FFT of the signal for each region highlighted in (**b**) including three wave packets (P1, P2, P3).

To numerically solve Eq. (1) for each *n* in the spin-chain, we developed our own program which was written in C++. This implements a second-order Runga–Kutta (RK2) midpoint method^41^ with a stepsize of $\delta t = 2.5 \times 10^{-15}\text {~s}$ across all simulations. We validated our RK2 results against a fourth-order Runga–Kutta (RK4) method and found, at such small $\delta t$ values, the RK4 method returns negligible differences compared to the RK2 method alongside incurring significantly greater computational costs. Our material parameters are $\gamma / 2 \pi = 28.8 \text { GHz} \cdot \text {T}^{-1}$ and $\alpha = 10^{-4}$^19^. We use an external static magnetic field $\mu _0 H_0 = 0.1 \text { T}$, and an exchange stiffness $D = 5.3 \times 10^{-17}~\text {Tm}^2$^42^. This gives an exchange interaction constant $J = 132.5~\text {T}$ ($53 \text { pJ/m}$), which can be obtained from $J = D/a^2$^43^, for a lattice constant $a = 6.32~\text {\text{\AA }}$; between that of YIG^44^ and pure iron^45^. With the number of sites in our structure, *N*, given by $N=15,811$ which results in our model corresponding to a physical length of $10{\upmu }$.

The purview of our model is to investigate the behaviour of spintronic devices, particularly looking at the response to the initiation of a signal. These are often driven and measured using line antennae, as shown in Fig. 1a, where a signal is generated by an antenna^46^ which we model this through $\pmb {h}_{\text {d}}$:

3 $$\begin{aligned} \pmb {h}_{d}(x,t)= h_0 \cos (2 \pi f_d t) \pmb {\hat{x}}, \end{aligned}$$

where $h_0$ is the oscillating driving field strength (taken as $\mu _0 h_0 = 3 \text { mT}$ throughout), and $f_d$ is the driving frequency. Using this, we uniformly drive 200 spins near one end of the chain, and record the local magnetisation response at $n = N/2$; corresponding to a second antenna placed $5~{\upmu }$ from the beginning of the driving region. To prevent backward reflections we implement absorbing boundary conditions (ABC)^47,48^ to ensure our spin waves behave as outgoing waves at the spin chain’s boundaries^49^. Each ABC is appended to both ends of the spin chain, and in each, $\alpha$ linearly increases from 10$^{-4}$ to 1 across 300 sites. We ensured that, regardless of the total time simulated, our sampling rate was far larger than the Nyquist frequency of the system^50^.

## Results

An example output is given in Fig. 1b for the time evolution of a signal of frequency $f_d = 15$ GHz which is applied transversely to the sample magnetisation for $5 \text { ns}$. We observe, when our signal is applied instantaneously, a propagating waveform with distinct features that can be grouped into three regions, as labelled in Fig. 1b: precursors, signal onset, and equilibrium. The precursor appears from the front of the wave train onward as a set of wave packets. These are ordered in increasing amplitude; each appears akin to its neighbours, but with variations in profile including amplitude and width. At 2.60 ns the final wave packet smoothly transitions, with a rapid increase in amplitude, into the second region. This constitutes the signal onset region. After a short interval which includes a dip in amplitude at 3.20 ns, the signal appears to stabilise into the equilibrium region.

**Fig. 2:**
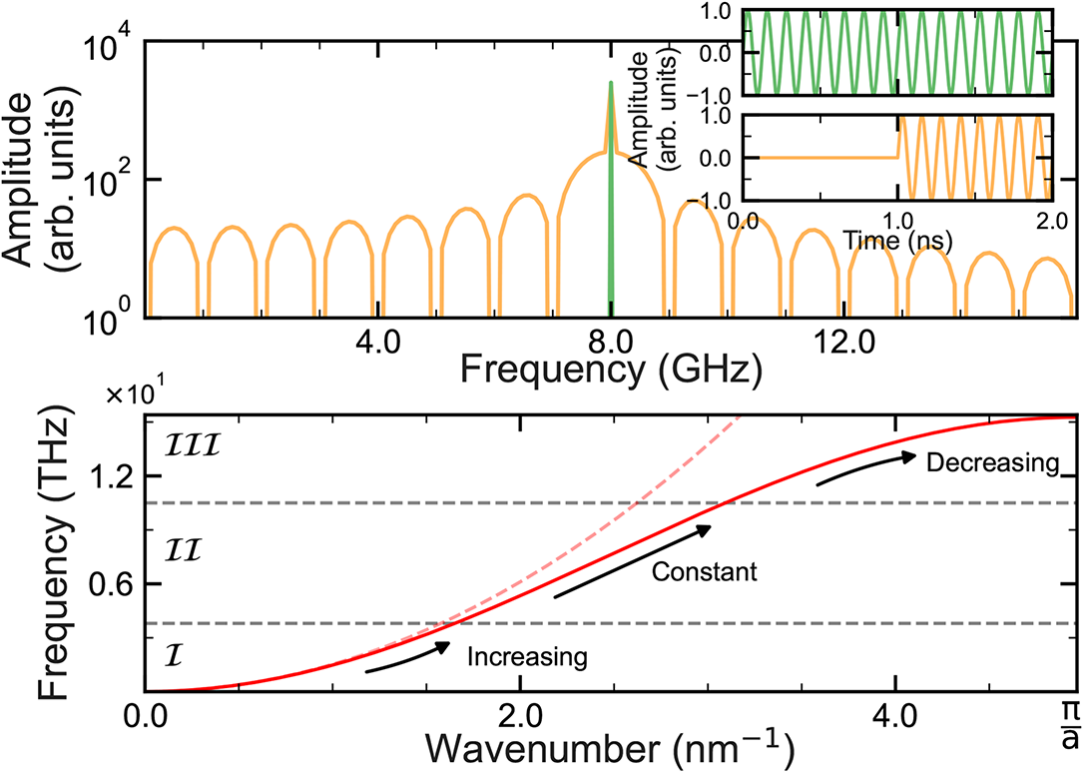
(**a**) Comparison between the FFT of quasi-Heaviside step function with no time delay (green) and a 1 ns delay (orange) as shown in the insets. Both signals terminate arbitrarily at 10 ns. (**b**) Dispersion relation for our system which has been separated into three thematic cases. These cases are based upon the rate of change of the group velocity, shown in Eq. (5). The dashed, red line represents $\omega = \gamma (H_0 + Dk^2)$ which is only valid in the limit $ka \ll 1$.

To illuminate the significance of, and understand, the regions identified in Fig. 1b we performed Fast Fourier Transforms (FFT) with results shown in Fig. 1c. These allow us to gain insight into the constituent frequencies present in each region. The FFT for all precursors displays several distinct regions. Analysing the FFTs for the individual wave packets (P1, P2, P3), shown as dashed lines, demonstrates that the earlier packets (P3) are composed of high frequencies relative to wave packets emerging later (P2, P1) which have lower constituent frequencies—all wave packets are still above the driving frequency. We note that the resolution of the spectra shown in Fig. 1c is best for P1 and poorer for successive wave packets This is due to the decreasing temporal widths that the wave packets span.

On the other hand, there are fewer key features in the FFT for the equilibrium and signal onset regions. These are largely centered around the driving frequency, $f_d$, with small contributions at lower frequencies. We surmise that the system transitions from a broad quasi-continuous range of frequencies in the precursors to a state of well-defined frequencies in the signal onset before becoming wholly dominated by $f_d$ in the equilibrium.

We have now identified all the features of the system, but we do not yet know their origins; this poses several questions. For example, it isn’t clear why the single driving frequency results in a multitude of quasi-discretised frequencies. Other questions include why it appears that each wave packet propagates at a different rate, and why the wave packets apparently disperse as the simulation progresses. To tackle these questions, we simplify our model by simply considering it to be a delayed sine wave as shown in the inset of Fig. 2a. This can be otherwise viewed as a quasi-Heaviside step function^51^. If we take the FFT of this signal, as seen in the main panel of Fig. 2b we get a well-defined main peak at the driving frequency surrounded by two wings: one for high frequencies, and one for low frequencies which are regularly spaced but of decreasing amplitudes. This example is an instance of a single driving frequency resulting in a continuous spectrum of output frequencies. Note that unlike the Heaviside step function example, in our system we can only resonantly excite spin waves at specific eigenfrequencies. For the ferromagnetic material parameters used here, these eigenfrequencies follow the dispersion relation as indicated by the solid red line in Fig. 2b. Furthermore, in both Figs. 1c and 2a, we don’t observe sharp peaks at individual frequencies. Instead, the broad frequency regions spanned by the wave packet’s FFT indicate a mixture of constituent frequencies. As the two systems strongly correspond, we deduce that each wave packet is a composite of frequencies which interfere with one another.

Taken together, this explains how a single $f_d$ can result in a multitude of output frequencies as wave packets, by stimulating a response at discrete eigenmodes permitted by coupling rules^40^. This also explains the emergence of wave packets in the precursors. The envelopes that each precursor wave packet form arise from interference between multitudes of frequencies that propagate at comparable velocities. For instance, signals generated between the front and rear portions of the driving region have a range of frequencies. The signals with the higher frequencies from the back region catch up with nearby, but lower, frequencies, from the front and interference to produce a wave packet. Initially the packets involve higher frequency waves which propagate faster under normal dispersion.

As time progresses, the packets involve lower frequency waves, (but still above the driving frequency), which have slower group velocities, but because they are composed of frequencies closer to the driving frequency these frequencies are more strongly excited as indicated by Fig. 2a. Further details on this, alongside the effect of varying the driving region width upon the precursors, is further discussed in Supplementary Note S2.

**Fig. 3:**
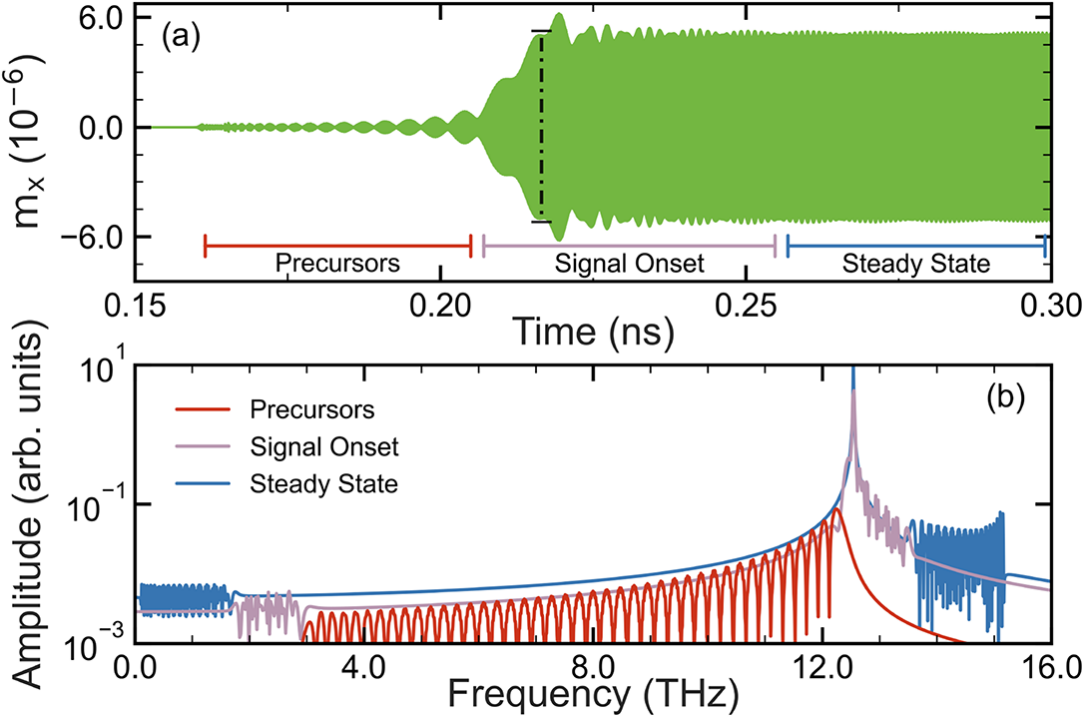
(**a**) Time evolution of a signal driven at $f_d=$ 12.54 THz, where the dashed line indicates the the arrival time for a signal with frequency $f_d$, and (**b**) FFT of the signal where the precursors can be seen to lie to the left of $f_d$.

We now examine how the development of the signal onset and precursors depends on the dispersion relation shown in Fig. 2b. In the low frequency limit^52^ the dispersion relation can be approximated to

4 $$\begin{aligned} \omega = \mu _0 |\gamma | (H_0 + Dk^2), \end{aligned}$$

which leads to the group velocity, $v_g$, through

5 $$\begin{aligned} v_g = \frac{d \omega }{d k} = 2 \mu _0 |\gamma | D k. \end{aligned}$$

Thus, from Eq. (5), the early wave packets (P1, P2, P3) in Fig. 1b that precede the signal onset should be comprised of frequencies that are higher than the driving frequency. This would lead to faster group velocities for the higher frequencies according to Eq. (5). This is all in agreement with the FFT data of Fig. 1c.

To illustrate how these findings can be more broadly applied to different dispersion relations, we now turn our attention to the high frequency limit [labeled as Case III in Fig. 2b] where the slope of the dispersion relation decreases with wave vector; in contrast to what has been discussed up to this point for the case of low frequencies where Eq. (4) is valid. We see, as shown in Fig. 3a, that the time evolution of the received signal resembles that shown in Fig. 1c. The propagation time is shorter, but the three distinct regions can be observed: precursors, signal onset, and equilibrium. When we examine the FFT in Fig. 3b, we see however, that while the global maxima lies at the driven frequency $f_d = 12.54$ THz, the frequencies contained in the wave packets of the precursor region in Fig. 3a are now *lower* than $f_d$.

In Fig. 1b, the wave packets are ordered such that the highest frequencies are furthest from the signal onset; they precede the main signal. Meanwhile, at the higher $f_d$ used in Fig. 3b, the inverse is true, where wave packets comprised of frequencies lower than $f_d$ are seen racing ahead of the equilibrium. This is a direct consequence of the slope inversion of the spin wave dispersion on $v_g$ for these two regions. They are also direct analogues to high-frequency (Case I) and low-frequency (Case III) Sommerfeld precursors^34,36^.

**Fig. 4:**
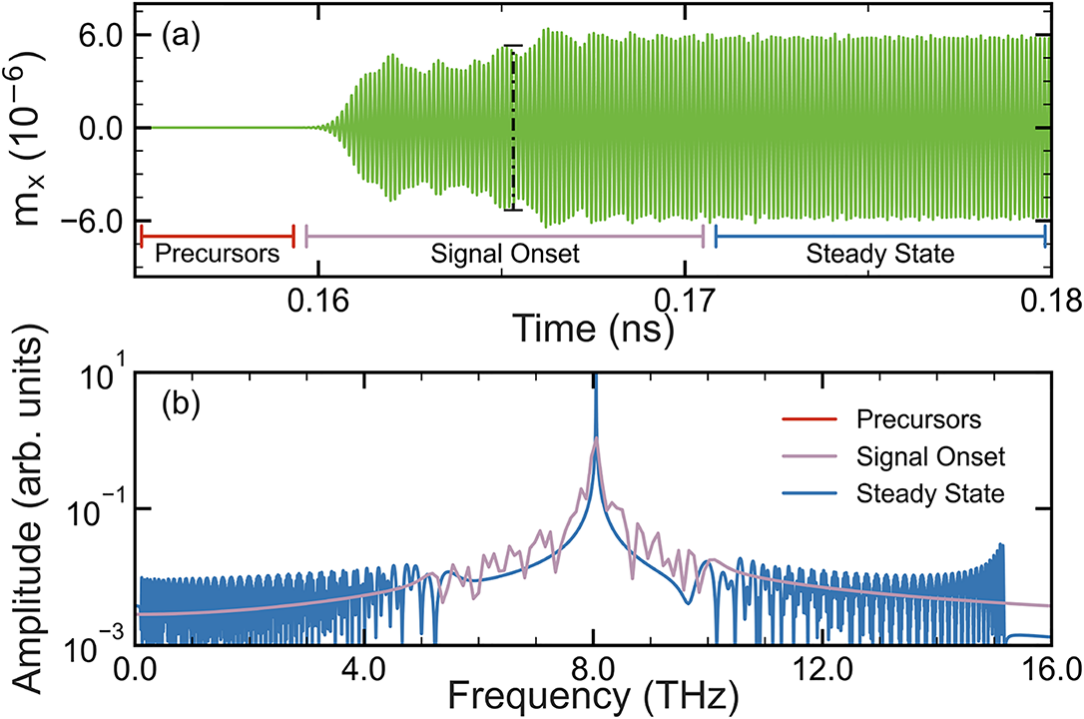
(**a**) Time evolution of a signal at $f_d= 8.05 \text { THz}$, where the dashed line indicates the the arrival time for a signal with frequency $f_d$, and (**b**) FFT of the signal where the precursors have negligible amplitude and the signal onset region is split and symmetric around $f_d$, similar to the steady state region.

Knowing that the slope of the spin wave dispersion plays a significant role on the decoherence shown here in the form of precursor pulses, we now turn to the response of signal driven at $f_d = 8.05 \text { THz}$ (Case II) where slope of the dispersion relation is constant. The results are shown in Fig. 4a, where, in stark contrast to the other two cases, there are no precursors. The reason for this is the linear dispersion relation so that all adjacent eigenmodes to our $f_d$ are travelling at the same $v_g$.

This means that instead of observing an oscillatory-train of wave packets, we instead observe a modulating signal beyond the initial amplitude increase; the frequencies within the equilibrium region here then disperse as a trailing wavetrain (Additional simulations shown in the supplemental material show that in Case II one can obtain rectangular waves if $f_d$ is applied for a finite time; terminated at both ends.). The corresponding FFT is shown in Fig. 4b and it is also drastically different to the other cases in that the shock wave and equilibrium regions both have global maxima at $f_d$; clearly demonstrating how the shape of the dispersion relation underpins intrinsic decoherence generation. Note that in Figs. 3 and 4$\alpha = 10^{-6}$ as in Eq. (1) the damping term scales with frequency^53^.

## Discussion

What has been discussed so far clearly indicates that dispersive decoherence from the linear oscillatory wave trains, reminiscent of DSW wave trains, are interference derivable phenomena, and are inherent to dispersive systems. Although this has been well established in electromagnetism^36,54^, somewhat similar phenomena have been recently shown to be present in YIG films, albeit associated to DSW^19,55^ and using nonlinear Schrodinger equation (NLS) model to motivate and explain the experimental results. The NLS model predicts that abrupt changes in the amplitude of a carrier frequency result in DSW, so long as the nonlinear components of a wave train dominate the effects of dispersion, while dissipation remains negligible^32^. In contrast, here we use a simple model; a one-dimensional magnetic spin chain where only Zeeman and exchange interactions are present, to show how precursors and their associate behaviour are an inherent property of magnetic systems. Not only that, but we also use very small excitations to generate the spin waves. In which case, any non-linear components of the generated signal will thus be small in comparison to dispersion and exchange effects. This collectively means that the spin motion remains strictly in the linear limit, and that the consequent decoherence from the generation of expanding wave trains does not necessarily require nonlinear behaviours.

It is a primary motivation in spintronics to design systems which transmit information quicker, and in denser volumes, than their electronic counterparts^56–58^; as shown here, this can be achieved using high-frequency excitations. However, most current spintronic devices are YIG-based and operate in lower frequencies of Case I of Fig. 2b, due to the large $\mathbf {H_0}$ that are required to operate beyond this^1^. Therefore, the significance of our results using high-frequency drives lies in understanding the features of spin wave decoherence. By doing so, we shed light on curious phenomena such as the intrinsic suppression of decoherence when the dispersion relation approaches a linear regime (case II), in addition to how the nature of forerunners can be reversed as the slope of the dispersion changes.

It is noteworthy that while we only considered a bulk FM spin wave dispersion relation, our findings should be transferable to propagation described by other dispersion relations. For example, in recent works^19^ which used thin film YIG samples, propagation was described by a surface spin wave dispersion relation. The work shows a behaviours similar to what we have in Case III, but at low frequency (6 GHz), where decoherence is still observed. Furthermore, even in ferromagnets, one doesn’t have to be at high wavevectors to have a linear dispersion. There are cases where dipolar and exchange contributions balance one another, leading to a linear dispersion even at small wavevectors^59^. Moreover, our finding for Case II should be applicable to systems such as antiferromagnets (AFM), as those can have linear dispersion relations^52,60,61^. Therefore, since AFMs have resonances often in the hundreds of GHz^61^, they present an excellent opportunity for not only engineering spintronic devices that could support decoherence free spin wave propagation, but they would also operate at higher frequencies than FM systems.

## Conclusion

We have demonstrated how dispersive decoherence is an inherent property of signals propagating through spin wave systems. Our model, which relies only upon Zeeman and exchange interactions, is an efficient way to study such effects. It produces features, akin in several ways to DSWs, that depend on the shape of the spin wave dispersion relation. These features are a result of interference that arise between two or more eigenmodes of differing group velocities. Such decoherence could be detrimental to magnonic devices possessing binary states, as their non-negligible amplitudes could constitute a third state while the transition between no signal and a steady state may not be sufficiently brief to allow for transistor applications. We find that decoherence control can be achieved through the spin wave dispersion relation such as decoherence suppression where the dispersion relation approaches a linear regime.

## Supplementary Information

Below is the link to the electronic supplementary material.

Supplementary Information.

## Data Availability

The datasets used and/or analysed during the current study are available from the corresponding author upon reasonable request.
