# Supplementary material for: Decoherence in spin wave propagation via precursor pulses during signal equilibration
Source: Sci Rep. 2025 Feb 6;15:4458. doi: 10.1038/s41598-025-88799-3 (PMC11802918; doi:10.1038/s41598-025-88799-3)
Supplement: Supplementary file 1 — Supplementary Information. [file 41598_2025_88799_MOESM1_ESM.pdf]

Supplementary Note S1. Negligible effect of dipole-dipole interaction

A core proposition of this work is that a simple simulation model is sufficient to recover behaviours and characteristics that are similar (and have historically been associated) to dispersive shock waves.

It is well accepted that at high frequencies the impact of dipolar fields H_{dip} would be negligible as the system is exchange-dominated. We would therefore expect that H_{dip} would exert the greatest influence at the lower-valued eigenfrequencies of our system (Case I). To investigate this, we performed simulations for the same system shown in Fig. 1(b), both with and without H_{dip}. These results can be seen in Supplementary Fig. 1. The red (background) signal has the same H_{eff} as in Eq. (2), while the green (foreground) signal includes a H_{dip} term accounting for dipole interaction between m_n and all other sites in the chain. The driving region is located between 0.1875 - 0.3125 µm; left of this region are our absorbing boundary conditions. We note a small difference in amplitude of the signal although the wavelength and main characteristics are unchanged. Overall, the two cases are in excellent agreement. Thus, we can surmise that the wave packets' profile and characteristics are resultant from the other mechanisms and interactions within our model. This data was recorded between 0 - 2.25 µm, instead across the full 10 µm length of our system, as the difference between the two signals of Supplementary Fig. 1 becomes negligible once both signals propagate further away from the driving region.

We should note however, that throughout this work we have considered the full dispersion relation and not just the low frequency limit. Cases II and III are well into the THz frequencies where not only has the slope of the dispersion relation changed, but this is also well beyond the long-wavelength limit; hence why we use a simple, but atomistic model without dipole-dipole interaction.

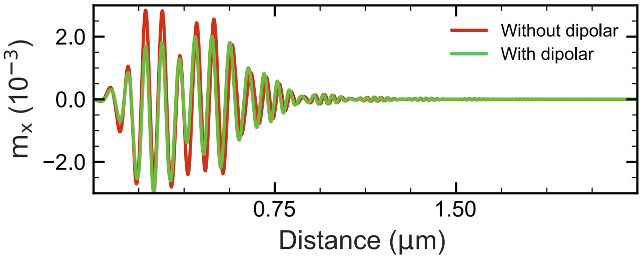

Supplementary Figure 1. Comparison of two signals both being driven at $f_d = 15$ GHz where one (green) has dipole-dipole coupling while the other (red) does not.

Supplementary Note S2. Driving region and precursor profiles

The envelopes observed are a consequence of interference between the different frequencies generated at different positions due to a finite driving region, or antenna as would be implemented in mangnonic devices. One can imagine this - in its most simplistic form - as two spins being drives at sites $n = 1$ and $n = 200$ as shown in the figure below; as opposed to the continuous driving region that is 200 sites in length as shown in the main manuscript. The faster propagating excitations generated at $n = 1$ will eventually catch up, and ultimately constructively and destructively interfere with the slower excitations originating at $n = 200$ at various positions along the spin chain. Thus, creating the wavepacket envelopes that we observe as shown in Supplementary Fig. 2.

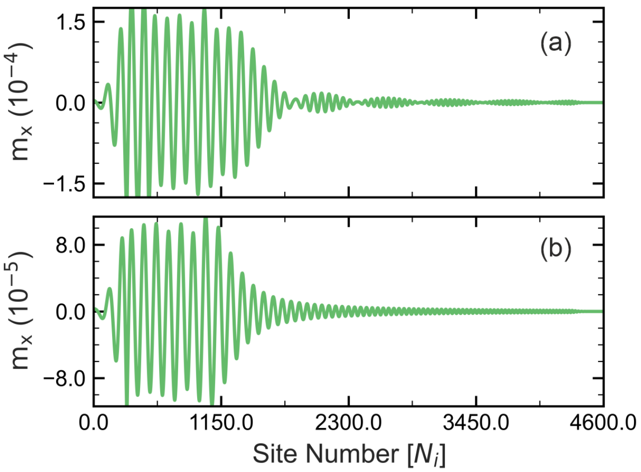

Supplementary Figure 2. (a) Two sites driven at positions $i = 1$ and $i = 200$ and $f_d = 15$ GHz. Wave packets can be seen to the right of the signal onset. (b) Only site $i = 1$ driven at $f_d = 15$ GHz. No wave packets can be observed.

Supplementary Note S3. Sharpness of the rising edge of the signal onset

In experimental setups, there is an elapsed time before the oscillatory pumping field $\boldsymbol{h}_d$ reaches its maximum strength of h_0; unlike in simulations where this can be achieved instantaneously. This time is called the rising time or time gradient t_g. We set our simulation to use the same parameters those of Fig. 1(b). We then introduced t_g by linearly increasing the oscillating pumping field strength at each timestep h in the simulation until it reached the value of h_0.

In both Supplementary Fig. 3(a) and Supplementary Fig. 3(b), we observe an overall decrease in the amplitude and prominence of the precursors as t_g increases. The steady-state region also contains less fluctuation in the maxima of individual oscillations as t_g increases. Finally, the steepness of the rising edge of the signal onset lessens as t_g increases. In Supplementary Fig. 3(a), there has not been sufficient time during propagation for the divergence of the precursors to become apparent. This indicates that altering t_g simply delays the arrival of the signal onset. However, in Supplementary Fig. 3(b), the delay in the arrival of the signal onset is accompanied with a decreased presence of the precursors. This therefore shows that varying the value of t_g offers a clear mechanism for mitigating the presence of precursors, but comes at the cost of increasing the system's response time.

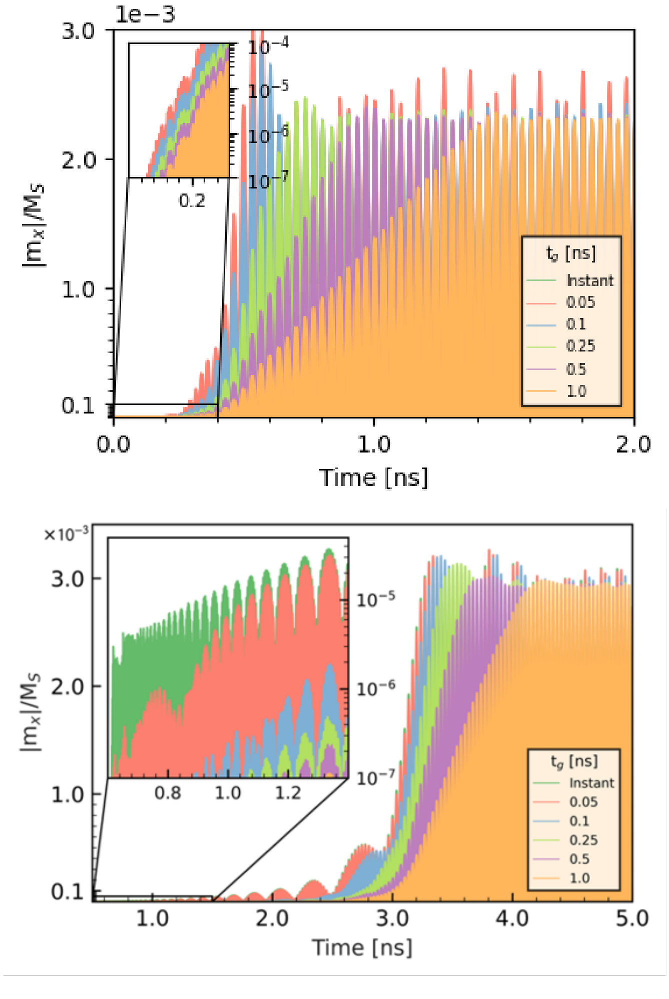

Supplementary Figure 3. Gradient time t_g measured at a distance of (a; top) 1 μm and (b; bottom) 5μm from the rightmost edge of the driving region.
